# Outcome Measure Utilization Demonstrates No Relation to Journal Rank or Level of Evidence for Superior Labral Tears of the Shoulder

**DOI:** 10.7759/cureus.7189

**Published:** 2020-03-05

**Authors:** Brendan A Williams, Ralph Michel, Gregory Y LaChaud, Daniel Weltsch, Kevin W Farmer, Joseph King

**Affiliations:** 1 Orthopaedic Surgery, Children's Hospital of Philadelphia, Philadelphia, USA; 2 Anesthesiology, University of Florida, Gainesville, USA; 3 Orthopaedics and Rehabilitation, University of Florida, Gainesville, USA; 4 Orthopaedic Surgery, The Chaim Sheba Medical Center at Tel Hashomer (Affiliated with the Sackler Faculty of Medicine), Tel Aviv, ISR

**Keywords:** anterior to posterior tear, clinician-measured outcomes, journal rank, level of evidence, patient-reported outcomes, superior labrum

## Abstract

Introduction

Superior labrum anterior to posterior (SLAP) tears are injuries of the shoulder's glenoid labrum involving biceps tendon insertion. We describe the scope of outcome measures used in the SLAP tear literature over the last decade and evaluate whether objective study metrics relate to level of evidence (LOE) and journal rank.

Methods

A comprehensive review of the literature was performed to identify all articles on the outcomes of the treatment of patients with SLAP tears published over the period of a decade (2007-2016). Studies were reviewed for LOE, SCImago Scientific Journal Ranking (SJR), study characteristics, and patient demographics. The utilization frequencies of outcome measures were recorded. Outcome measures were categorized as clinician-measured outcomes (CMOs), outcome scores/patient-reported outcomes (PROs), or other reported outcomes (OROs). Univariate analyses compared demographics and outcome-measure utilization among studies grouped by LOE (high: I/II vs. low: III/IV) and SJR (high: >2 vs. low: <2).

Results

The literature review identified 86 studies meeting inclusion criteria from an initial search of 582 articles. The mean study SJR was 2.02 and 0.85 +/- 0.79 CMOs were included. Twenty different outcome scores were utilized in the reviewed literature with a mean of 2.66 +/- 1.61 PROs/study. The most common outcome scores were ASES score, pain Visual Analog Scale (VAS), and constant score. High SJR studies had longer follow-up (p=0.036). High LOE studies utilized more CMOs (p=0.008) and more commonly included physical exam findings (p=0.0015). Revision surgery rate was less commonly reported in high LOE studies (p=0.036). None of the other outcome measures studied were associated with high study LOE or SJR.

Conclusions

Considerable variability exists in outcome-measure utilization within the recent SLAP tear literature. ASES score, pain VAS, and constant score are the most commonly reported PROs. CMOs and OROs are infrequently reported. There is little objectivity distinguishing higher and lower quality studies based on the proxy metrics used. Future SLAP tear research should focus on improving the consistency of outcome-measure reporting to enable more meaningful cross-study comparisons.

## Introduction

Awareness and interest in the pathoanatomy and management of superior labral tears of the shoulder anterior to posterior (SLAP) have grown since their first description and early classification in the late 20th century [1-2]. Coincident with this trend has been the evolution of measures of patient outcomes within the orthopedic literature. Basic clinical and radiographic findings are no longer a sufficient barometer of success. Orthopedics has widely adopted additional outcome tools focused on the patient and his or her individual response to treatment: patient-reported outcomes (PROs) [3]. Patient-reported measures and outcome scores have become a fundamental component of outcome studies and a cornerstone of high-level research, adding to the vast array of traditional measures such as range of motion (ROM), return-to-work/sport rate, and complication rates. 

With the breadth of available measures and instruments and without the standardization of use, variability in outcome-measure reporting can make meaningful cross-study comparison difficult and lead to inconsistent interpretations of the literature [4-6]. Such variability has been well-documented in other highly studied areas of orthopedics such as rotator cuff repairs and anterior cruciate ligament injuries and, more recently, within the SLAP tear literature [7-9]. However, what remains unclear is whether the breadth and depth of outcome-measure reporting relate to a study’s potential to affect change in clinical practice. Level of evidence (LOE) and the rank of the journal in which a study is published serve as easily accessible metrics that are likely related to the impactfulness of a given study. Thus, determining whether they hold any association with outcome-measure utilization is of interest in answering this lingering question.

The aim of this study was to rigorously evaluate the outcome-based SLAP tear literature over the past decade in order to aid in navigating future research efforts through better-informed study design and selection of outcome measures. Our primary purposes were the following: (1) to describe the scope of outcome measures utilization throughout the SLAP tear literature over the last decade; and (2) to evaluate whether objective study metrics and outcome-measure usage relate to proxy quality measures of research: LOE and journal impact factor/scientific journal rank (SJR). We hypothesized that greater outcome-measure utilization would be seen in a higher level of evidence studies and those published in higher-ranked journals.

## Materials and methods

A comprehensive, systematic literature review was performed of the PubMed and Cochrane databases, spanning a decade-long period, identifying SLAP tear outcome studies published over a decade from January 1, 2007, to December 31, 2016. The keywords used for the database searches were “shoulder,” “SLAP,” “superior labrum,” and “superior labral.” Inclusion criteria consisted of outcome studies, including patients with superior labral pathology of any type. Studies containing patients with concomitant pathology and comparison groups with other pathology were also included if the percentage of patients with superior labral pathology was greater than 20%. This cutoff was selected in order to be maximally inclusive of studies while still examining literature where the management of patients with SLAP tears was a significant component. Exclusion criteria included review articles, systematic reviews, meta-analyses, case reports, surgical technique articles, and studies without full text available in English.

The title and abstract review was completed for all articles found in the primary database searches. The screening was completed by two authors. Articles were selected for a full-text review according to the aforementioned inclusion and exclusion criteria. The full-text review was then used to identify those matching study parameters. As additional cross-referencing, a hand search was performed of the works cited of 13 narrative and systematic review articles identified during our literature search. These were surveyed by title, abstract, and full-text review for additional studies eligible for inclusion. Two or more authors participated in all phases of the review process and data extraction. Consensus over any discrepancies was reached by discussion and mutual agreement between the first and senior authors.

Included studies were reviewed for LOE, SCImago Scientific Journal Ranking (SJR), study characteristics, as well as subject and labral pathology demographics. All outcome measures that were reported in patient follow-ups were documented and grouped as clinician-measured outcomes (CMOs), PROs, and other reported outcomes (OROs). Measures including a clinician-measured component in the scoring (e.g., the American Shoulder and Elbow Surgeons (ASES), Constant, and the University of California at Los Angeles (UCLA) scores) were grouped as PROs, as these are a form of outcome scores. Among the PROs, clearly defined and/or previously validated outcome measures were tallied individually. However, multiple studies included PRO measures for pain and activity that were described in detail in the studies even if they were not routinely used in the literature or validated scores. These scores were grouped and tallied separately based on their area of assessment as “not otherwise specified (NOS).” All collected study variables and identified outcome measures are summarized in Table 1. Bias (as per the Preferred Reporting Items for Systematic Reviews and Meta-Analyses (PRISMA) guidelines) was not included in the assessment because it was not relevant to the purpose of this study, which examined the reporting of outcome measures and did not compare the results of the measures themselves [10].

**Table 1:** Comprehensive list of collected study variables and outcome measures identified *CMO, clinician-measured outcomes; PRO, patient-reported outcomes; ORO, other reported outcomes; SJR, SCImago scientific journal ranking; LOE, level of evidence; ASES, American Shoulder and Elbow Surgeon Standardized Shoulder Assessment Form; DASH, Disability of the Arm, Shoulder, and Hand; EuroQoL, European Quality of Life; ISIS, Instability Severity Index Score; KJOC, Kerlan-Jobe Score; LHB, Long Head of Biceps Score; SANE, Single Assessment Numeric Evaluation; SSV, Subjective Shoulder Value; SF-12, Short Form-12; SF-36, Short Form-36; SRQ, Shoulder Rating Questionnaire; SST-12, Simple Shoulder Test; VR-36, Veterans RAND 36 Item Health Survey; UCLA, University of California, Los Angeles Shoulder Score; VAS, visual analog scale; WOSI, Western Ontario Shoulder Instability Index; NOS, not otherwise specified; ROM, range of motion; SLAP, superior labrum anterior to posterior

Study Variables	Outcome Measures
Journal and Study Characteristics	Journal of Publication, SJR, LOE
Study Demographics	Number of subjects, Length of follow-up, Mean patient age, Gender distribution
CMOs	Forward elevation/flexion, Abduction, External rotation, Internal rotation, External rotation at 90 degrees, Internal rotation at 90 degrees, Other physical exam findings, ROM score NOS, Strength score/assessment NOS
PROs	ASES, Constant or Normalized Constant, DASH, EuroQol, ISIS, KJOC, LHB Score, Oxford Instability Score, Quick DASH, Rowe Score, SANE, SSV, SF-12, SF-36, SRQ/L Insalata Shoulder Questionnaire, SST-12, VR-36, UCLA, VAS Pain, Walch Duplar Instability Score, WOSI, Activity/Functional Score NOS, Pain Score NOS Other Patient-Reported Score/Measure NOS
OROs	Return to Work, Return to Activity/Sport, Complication Rate, Revision Surgery Rate, SLAP Re-tear Rate, Satisfaction

Individual ROM assessments were only documented as included if post-treatment degree values or scores were reported. ROM assessments that did not include degree measurements were tallied separately as “not otherwise specified (NOS).” Strength assessments and other physical exam findings (e.g., tenderness, signs, or maneuvers) were included if the presence or absence was reported in patient follow-up.

For articles not clearly designating LOE, the level was derived from the Journal of Bone & Joint Surgery LOE standards and confirmed by two authors [11]. The SJR was obtained for each journal once all included articles had been identified [11-12].

Statistical analyses were performed using JMP® PRO Version 12.0.1 (SAS Institute, Cary, NC). Descriptive statistics were used to analyze the gathered study variables and the inclusion frequency of each of the outcome measures gathered (individually and grouped). In order to assess for study bias based on the inclusion of studies that were not exclusively examining patients with superior labral pathology, a subgroup analysis was performed to compare the studied variables among isolated SLAP tear studies and those including other pathology. Univariate analyses were then performed comparing study demographics and outcome-measure utilization among studies grouped by LOE (high: I/II vs. low: III/IV) and SJR (high: >2 vs. low: <2). Continuous variables were compared using the student t-test while nominal variables were compared with chi-square testing. A p-value of <0.05 was considered significant.

## Results

Study and patient demographics

**Figure 1:**
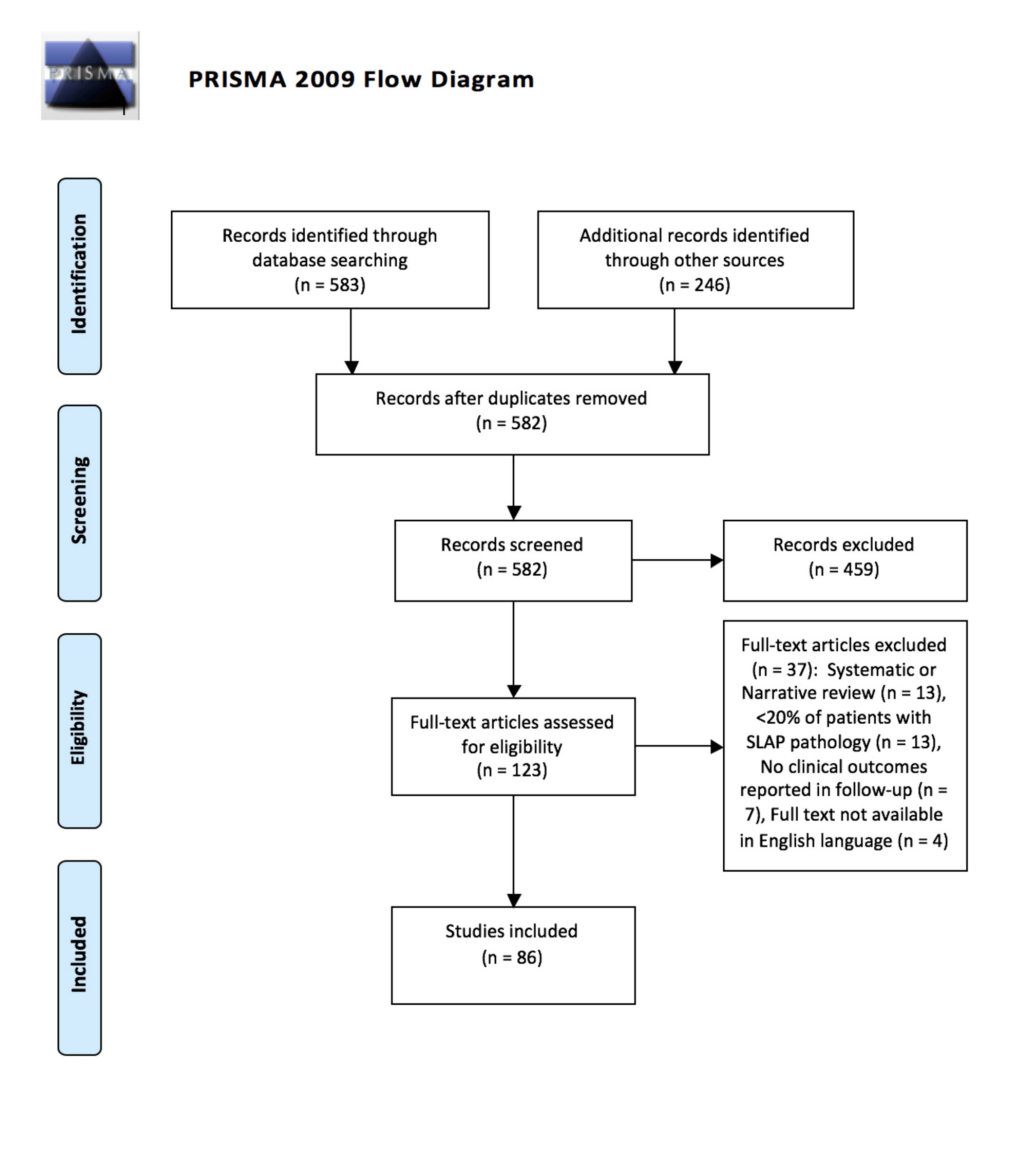
PRISMA flow diagram The PRISMA flow diagram of the comprehensive literature review performed for study identification using the methodology of systematic reviews PRISMA: Preferred Reporting Items for Systematic Reviews and Meta-Analyses

**Figure 2:**
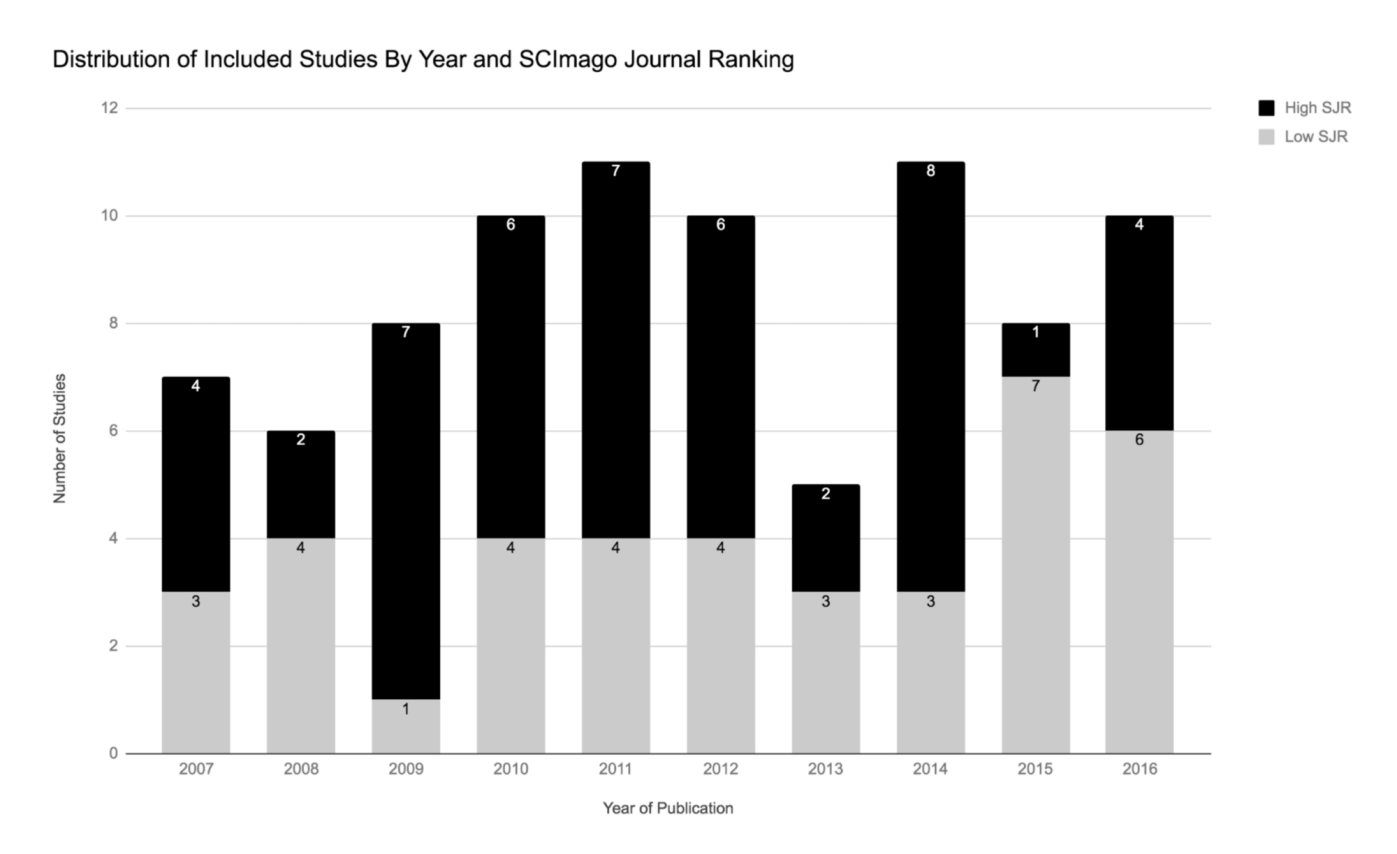
Distribution of included studies by year and SCImago journal ranking

**Figure 3:**
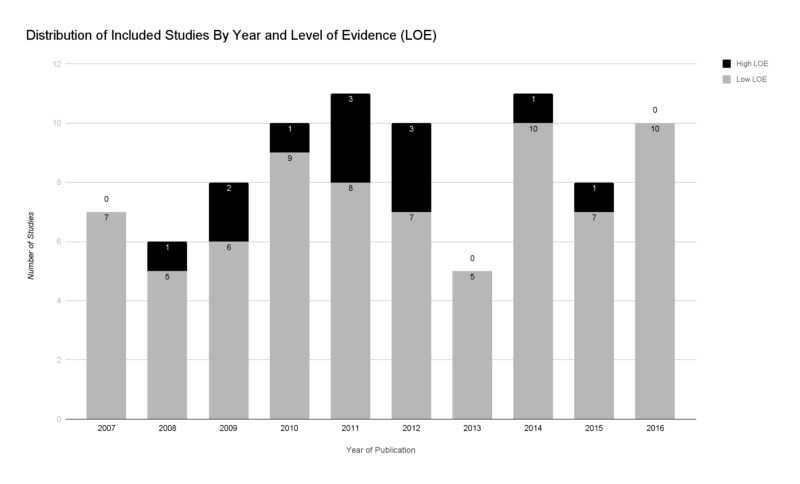
Distribution of included studies by year and level of evidence

The initial search identified 582 unique articles, of which 86 remained after the completion of our systematic-style comprehensive review over a decade-long period (Figure 1). Included studies were published in 24 different journals, the most common being the American Journal of Sports Medicine (28), Knee Surgery Sports Traumatology Arthroscopy (10), Arthroscopy (9), and the Journal of Shoulder and Elbow Surgery (7). Studies originated from 17 different countries, most frequently being the United States (43). Isolated SLAP tears were the focus of 39.5% of studies. Worker’s compensation status among included patients was not documented in 68.6% of studies. Of studies documenting worker’s compensation status (N = 27), 19 (70.4%) included this patient population among the study cohort. Study demographics are further detailed in Table 2. Subgroup comparison of studies of isolated SLAP tears and those including other shoulder pathology demonstrated no difference between study demographic variables and reporting of all outcome measures except for SLAP re-tear rates (38.2% vs. 17.3%, p = 0.03). The distribution of studies by year based on SJR and LOE can be seen in Figures 2-3, respectively. No significant temporal trend in publication frequency was noted in the reviewed decade of literature.

**Table 2:** Frequency of selected outcome measure reporting among reviewed studies* *CMO, clinician-measured outcomes; PRO, patient-reported outcomes; ORO, other reported outcomes; ASES, American Shoulder and Elbow Surgeon Standardized Shoulder Assessment Form; SANE, single assessment numeric evaluation; SSV, subjective shoulder value; SST, simple shoulder test; UCLA, University of California, Los Angeles Shoulder Score; VAS, visual analog scale; ROM, range of motion; SLAP, superior labrum anterior to posterior

Study Variable	Mean/Median* (range and/or percentage)
SJR	2.02 (0.12-3.41)
LOE	
Level 1	3 (2.4%)
Level II	9 (10.6%)
Level III	30 (35.3%)
Level IV	44 (51.8%)
Patient Age (Study Mean)	37.3 (19.1-65.5)
Number of Slap Tears	29.5* (6-4975)
Follow-up - Months (N = 84)	31.9 (3-85)
Percent of Female Patients	22.8% (0%-73%)
Study Included only Isolated SLAP Tears	
Yes	34 (39.5%)
No	52 (60.5%)
Study Included Worker’s Compensation Patients	
Yes	19 (22.1%)
No	8 (9.3%)
Not Documented	59 (68.6%)
Management of SLAP Tears	
Surgical	82 (95.3%)
Non-operative	2 (2.3%)
Combined	2 (2.3%)
Surgical Treatment - SLAP Repair	
Yes	73 (84.9%)
No (Debridement/Tenotomy or Tenodesis)	11 (12.8%)
Not Documented	2 (2.3%)

Outcome-measure reporting

A mean of 0.85+/-0.79 CMOs was included in each of the reviewed studies, with 60.5% of studies including at least one CMO. Range-of-motion assessments were included in less than half of studies, with forward flexion/elevation being the most commonly reported motion assessment. PROs were included in 93% of studies with an average of 2.66+/-1.61 PROs per study. Twenty different PROs were utilized in the reviewed literature; however, 13 of these were used in less than 10% of studies. The most common outcome scores used were American Shoulder and Elbow Surgeon Standardized Shoulder Assessment Form (ASES) (59.3%), VAS-Pain (41.9%), and Constant score (32.6%). Return to activity/sport was the most commonly included ORO. SLAP re-tear rates were reported in only roughly one-quarter of the studies, but re-tears were infrequently documented as being confirmed on advanced imaging (CT or MRI). Inclusion frequencies of the commonly reported CMOs, PROs, and OROs are detailed in Table 3.

**Table 3:** Frequency of selected outcome measure reporting among reviewed studies* *CMO, clinician-measured outcomes; PRO, patient-reported outcomes; ORO, other reported outcomes; ASES, American Shoulder and Elbow Surgeon Standardized Shoulder Assessment Form; SANE, single assessment numeric evaluation; SSV, subjective shoulder value; SST, simple shoulder test; UCLA, University of California, Los Angeles Shoulder Score; VAS, visual analog scale; ROM, range of motion; SLAP, superior labrum anterior to posterior

Outcome Measure	Percent of Studies Including
CMOs	
ROM Assessment (Any)	48.8%
ROM Degree Measurement	46.5%
Forward Flexion/Forward Elevation	40.7%
Abduction	14.0%
Internal Rotation	20.9%
External Rotation	34.9%
Internal Rotation @ 90 degrees	15.1%
External Rotation @ 90 degrees	23.3%
Physical Exam Findings	25.6%
Strength Assessment	10.5%
PROs	
ASES	59.3%
VAS Pain	41.9%
Constant	32.6%
SST	24.4%
UCLA	23.3%
SANE/SSV	18.6%
Rowe Score	11.6%
OROs	
Return-to-activity/sport Rate	51.2%
Return-to-work Rate	23.3%
Complication Rate	41.9%
Revision Surgery Rate	37.2%
SLAP Re-tear Rate (Imaging Confirmed)	25.6% (9.3%)

Quality trends in outcome reporting

In the univariate analysis, mean patient follow-up was found to be longer in high SJR studies (35.0 (95% CI: 30.0 - 40.0) vs. 28.3 (95% CI: 24.1 - 32.3) months, p=0.036). High LOE studies utilized more CMOs (1.50 (95% CI: 0.99 - 2.01) vs. 0.74 (95% CI: 0.57 - 0.92), p=0.008) and more commonly included physical exam findings in follow-up (66.6% vs. 18.9%, p = 0.0015). Revision surgery rate was less commonly reported in high LOE studies (41.9% vs. 9.1%, p=0.036). Higher SJR and LOE were not associated with the utilization of any other individual outcome measure (ORO, PRO, or CMO other than listed above) or the total number of PROs included.

## Discussion

The principal findings of this work demonstrate that studies of a higher level of evidence and those published in higher-ranked journals demonstrate little difference in outcome-measure utilization and reporting compared to other studies. Persistent variability in outcome-measure reporting was identified over the last decade in the SLAP tear literature in addition to the low reporting rates of critical measures of treatment success. This information highlights critical areas for improvement for any future researcher examining treatment options for this patient population.

This study identified that the vast majority of SLAP studies are of lower LOE (level III and IV), and use roughly three PROs for measuring patient outcomes, most commonly being ASES, VAS pain, and Constant scores [13-15]. CMOs are not consistently reported, with even the most common (ROM) described in fewer than 50% of studies. Other regularly reported outcomes, notably complication and revision surgery rates, are even less frequently reported. This lack of consistency makes interpretation and cross-study comparison within the literature difficult, restricting the meaningful evaluation of emerging and evolving treatment options in the management of SLAP tears of the shoulder.

Although the exploration of outcome-measure reporting practices within the orthopedic literature has become a popular topic recently, only one prior study has explored this issue in the realm of SLAP tears. Similar findings of variability were identified in the recent work by Steinhaus et al. with some overlap in the study period [9]. However, this work did not examine the relationship between outcome-measure utilization and other study metrics and instead focused more on return-to-play reporting. LOE was only briefly discussed and journal ranking/impact was not examined. Also of interest, during the overlapping time periods of the two studies available for comparison (2010-2014), the Steinhaus work identified 35 studies while our study included 47. This is likely due to the more lenient inclusion of studies on combined lesions and those including other shoulder pathology. It was also felt to be more representative of the entire SLAP literature, which often includes concomitant diagnoses. The present study confirms many of the findings of the Steinhaus work regarding the variability in outcome-measure utilization but in a larger and more recent cohort of studies. Additionally, it provides an in-depth exploration of LOE and journal ranking as it relates to the reporting of outcome measures. Our findings are unique in that this is the first study to our knowledge to examine outcome-measure reporting in the orthopedic literature as it relates to these proxy measures of study quality and impactfulness.

In selecting metrics of study quality, we sought to use objective, quantitative measures that were easily accessible and interpretable by the common orthopedist. While standardized methodologic quality checklists exist, they are impractical to use for the casual interaction with the medical literature [10,16-17]. Additionally, most physicians are not actively engaged in clinical research and are likely unlikely to be familiar with them. Thus, the quality and potential impact of a study is more commonly judged by more readily available parameters such as the LOE and the ranking of the journal of publication. These metrics, although less rigorous, carry tremendous implications for a study’s potential to influence the decision-making of the majority of physicians and ultimately alter the practice of orthopedics. We hypothesized that these higher-quality studies would have some objective differences in terms of study design, particularly in the realm of outcome-measure reporting. Interestingly, this was not the case. Studies from higher-ranked journals only demonstrated longer patient follow-up. Higher LOE studies did show some additional differences among CMO utilization and revision surgery rate reporting. However, in regards to outcome-measure reporting overall, we found little that distinguishes higher and lower quality studies based on the metrics utilized.

Based on our study findings, we have identified essential areas for improvement in the SLAP tear literature. First and foremost, we feel that reporting of complication rates and revision surgery rates is an essential component of any outcome study in the surgical literature. Reporting rates of these factors were dismally low in the literature reviewed, 41.9% and 37.2%, respectively. And similar to the findings of Steinhaus et al., return to activity/sports reporting also has substantial room for improvement, particularly for a pathology prevalent in an athletic population [9].

Another change that would be of tremendous benefit to the SLAP tear literature is a move toward standardizing the utilization of PRO measures. In our review of the past decade, we identified 20 different measures in circulation, only seven of which were reported in more than 10% of studies.

In a recent survey of surgeons in three large sports medicine and arthroscopy-focused organizations, Provencher et al. evaluated the use of outcomes instruments in clinical practice [18]. Consistent with the recent literature examined in this study, the ASES and Constant scores were among the three most preferred measures for the management of SLAP tear patients. The UCLA score was also a popular measure (second) among the surveyed surgeons for this patient population, whereas this score was the fifth most commonly utilized measure in the recent SLAP literature. Preference for these three scores was similar in the evaluation of rotator cuff and anterior instability patients, who are often present in cohorts examining SLAP tear outcomes. Establishing formal consensus and/or publisher guidelines for the use of these scores in future SLAP tear studies may assist in improving the quality and consistency of the literature base. Clear and structured outcome reporting of measures useful from a research and clinical care standpoint would enable meaningful cross-study comparisons and facilitate the progression of evidence-based patient care.

In a move towards this goal, Schmidt et al. examined the existing cohort of shoulder-specific PRO outcome measures objectively utilizing the Evaluating Measures of PROs (EMPRO) tool [3]. Their findings suggested that the ASES, SST, and Oxford Shoulder Score should be the primary options considered for measuring function and disability in patients with shoulder disorders. The Constant Score, of note, was not included in this analysis because aspects of this score require clinician response. Taking these findings into account along with the results of our study, investigators are empowered to select PROs for inclusion in future work that are reliable, valid, responsive, and have a basis for comparison within the existing literature. And although the Patient-Reported Outcomes Measurement Information System (PROMIS) has recently emerged within the orthopedic literature and carries the potential for creating a common language for outcomes reporting, limitations remain with respect to its implementation, and little evidence exists supporting its use in the athletic population [19]. Thus, it is likely too early to make this a standard measure for reporting, but researchers should stay attuned to its development as it may aid in improving the efficiency of shoulder outcome data collection in the near future.

Ultimately, journals may use this study and others to drive change in outcome reporting by setting publication standards. This is important not only within the shoulder literature but all orthopedic literature as the use of PRO measures expands. Journals often establish minimums of reporting for eligibility for publication (e.g., 2-year follow-up). Through structured evaluation in a manner similar to Schmidt et al., objectively selected essential sets of outcome measures could be instituted in popular areas of research [3].

Limitations

This study had several important limitations. First, despite performing a systematic review of two separate databases, other databases exist that were not included; thus, it was possible to have missed studies meeting study criteria. In a similar manner, exclusion of studies unavailable in the English literature may have biased our results towards outcome measures developed or more commonly utilized in English-speaking countries. Second, our utilization of the selected metrics of study quality in this manner has not been previously described and other important criteria for assessing study quality exist that were not examined before in our analysis [10,16-17]. Finally, our methods of selecting measures for inclusion introduce a source of potential variation from other similar works. Many studies may gather a considerable amount of patient data but limit their reporting to notable or significant outcome findings. We opted to only include a measure as “reported” if objective values were included in the results, i.e., stating that a measure was assessed in the methods alone was not sufficient for inclusion. True outcome-measure acquisition may also be different from what is reported in the literature.

## Conclusions

This study identified ongoing variability in outcome-measure reporting within the SLAP tear literature concurrent with prior investigations. Additionally, it was demonstrated that there is little objectively differentiating higher and lower quality studies in regard to outcome-measure reporting using the metrics described. Based on these findings, we recommend that SLAP investigators seek to standardize outcome-measure inclusion in future research efforts to enable better cross-study comparisons. Greater attention should be paid to the inclusion of CMOs, complication rates, and revision surgery rates. Ideally, standards for study design should be raised for publication in high-impact journals to drive this change.
